# Iterative feature removal yields highly discriminative pathways

**DOI:** 10.1186/1471-2164-14-832

**Published:** 2013-11-25

**Authors:** Stephen O’Hara, Kun Wang, Richard A Slayden, Alan R Schenkel, Greg Huber, Corey S O’Hern, Mark D Shattuck, Michael Kirby

**Affiliations:** 1 Department of Mathematics, Colorado State University, Fort Collins, CO, USA; 2 Department of Microbiology, Immunology, and Pathology, Colorado State University, Fort Collins, CO, USA; 3 Kavli Institute for Theoretical Physics, University of California, Santa Barbara, CA, USA; 4 Department of Mechanical Engineering & Materials Science, Department of Applied Physics, and Department of Physics, Yale University, New Haven, CT, USA; 5 Physics Department, The City College of New York, New York, NY, USA; 6 Department of Mechanical Engineering & Materials Science, Yale University, New Haven, CT, USA

**Keywords:** Feature selection, Microarray, Discrimination, Classification, Pathways, Sparse SVM, Influenza

## Abstract

**Background:**

We introduce Iterative Feature Removal (IFR) as an unbiased approach for selecting features with diagnostic capacity from large data sets. The algorithm is based on recently developed tools in machine learning that are driven by sparse feature selection goals. When applied to genomic data, our method is designed to identify genes that can provide deeper insight into complex interactions while remaining directly connected to diagnostic utility. We contrast this approach with the search for a minimal best set of discriminative genes, which can provide only an incomplete picture of the biological complexity.

**Results:**

Microarray data sets typically contain far more features (genes) than samples. For this type of data, we demonstrate that there are many equivalently-predictive subsets of genes. We iteratively train a classifier using features identified via a sparse support vector machine. At each iteration, we remove all the features that were previously selected. We found that we could iterate many times before a sustained drop in accuracy occurs, with each iteration removing approximately 30 genes from consideration. The classification accuracy on test data remains essentially flat even as hundreds of top-genes are removed.

Our method identifies *sets* of genes that are highly predictive, even when comprised of genes that individually are not. Through automated and manual analysis of the selected genes, we demonstrate that the selected features expose relevant pathways that other approaches would have missed.

**Conclusions:**

Our results challenge the paradigm of using feature selection techniques to design parsimonious classifiers from microarray and similar high-dimensional, small-sample-size data sets. The fact that there are many subsets of genes that work equally well to classify the data provides a strong counter-result to the notion that there is a small number of “top genes” that should be used to build classifiers. In our results, the best classifiers were formed using genes with limited univariate power, thus illustrating that deeper mining of features using multivariate techniques is important.

## Background

The sequencing of complete genomes has accelerated biomedical research by promising a greater degree of understanding of the biological processes encoded. The role of computational biology has become increasingly important because there is a significant need for progressing beyond statistics and data clustering. New analysis methods are needed that afford the ability to use more of the generated data, thus revealing more insights into biological processes. Mathematical optimization algorithms, and related machine learning techniques arising in computer science, have emerged as having significant potential for knowledge discovery in biological data sets arising in genomics, metabolomics and proteomics investigations. These methods are attractive given they are capable of identifying multivariate interactions associated with biological complexity, e.g., co-expressing genes in single or related pathways. Further, the gene selection procedure is data driven, thus providing an unbiased approach to knowledge discovery.

The emphasis on the search for *parsimonious models* – that is, models which attain high accuracy while minimizing the number of features – is now standard operating procedure in machine learning. Currently, many machine learning feature selection methods applied to microarray data explicitly eliminate genes which are redundant, in terms of discriminative or predictive value, with an existing set [1-8]. A recurring theme in this body of literature is to select a small set of “top ranked” genes. Parsimony leads to computational advantages when analyzing large-scale data and can improve the signal-to-noise ratio. However, when applying machine learning methods to biological data, the advantages of parsimony are less clear.

Microarray data typically includes highly correlated gene expression levels associated with a (potentially unknown) biological process. Yet feature selection methods tend to eliminate genes which fail to provide additional discriminatory power to the model. To the machine learning expert, this yields the desired parsimonious model. To the biologist, the suppression of potentially biologically-relevant information can impede process discovery.

We hypothesize that genes selected as most important to a parsimonious model are in fact only a small subset of a large set of highly discriminative, and thus informative, genes. To test this hypothesis, we employ a sparse Support Vector Machine to build an accurate model using only a small fraction of the features in the data. We then repeat this process, but at each iteration we remove all the genes selected by previous iterations from the data. At each iteration, the model must be built without the “most important” genes identified previously. We call this process Iterative Feature Removal (IFR).

If our hypothesis holds, there should be several iterations where the accuracy remains high, even as the top genes are iteratively removed from consideration. Eventually, the process will have removed all the relevant genes, and the accuracy should fall to random performance. Our results support this hypothesis. On the evaluated data sets, IFR demonstrates that at least 20 iterations are required before we see a significant loss in discriminatory power, providing hundreds of genes for further analysis.

## Results and discussion

### Data Sets

For this study, we examine four microarray data sets from the literature, as summarized in Table 1. The influenza data is from the Duke pan-viral challenge [9,10]. This data has gene expression levels taken from peripheral blood samples of human subjects exposed to H3N2 and H1N1 influenza. The H3N2 data consists of the gene expression levels of 12,023 genes collected from 17 human subjects in approximately 8-hour intervals. The H1N1 data measures the same set of genes, but on a separate set of 19 subjects, using the same collection methodology. In both H3N2 and H1N1, approximately half of the subjects became symptomatic, the others remained healthy.

**Table 1:** Overview of data sets

**Data set**	**Training samples**	**Testing samples**	**Features**	**Classes**
Influenza 14-16 [10]	51	57	12023	Asymptomatic, Symptomatic
Influenza 11-14 [10]	68	75	12023	Asymptomatic, Symptomatic
Lung Cancer [11]	32	149	12533	Mesothelioma, Adenocarcinoma
Prostate Cancer [12]	102	34	12600	Tumor, Normal
BCell Lymphoma [13]	47	–	4026	Germinal, Activated

Influenza 14-16 and 11-14 represent different temporal intervals of the data from [10]. The former is used to compare with other published results, and the latter is used because it provides more training samples for the automated analysis. All data sets except the BCell lymphoma have defined training and test partitions.

We use two subsets of the influenza data, labeled as “Influenza 14-16” and “Influenza 11-14” in the table. The first uses only those samples from time intervals 14 through 16, the second from intervals 11 through 14. The 14-16 data are used in [10], and are claimed to reflect peak symptom expression in the subjects. We employ this partition for the purposes of comparison with other published results. However, our analysis leads us to believe that intervals 11-14 represent the true period of peak symptom expression with the additional benefit of providing more samples for training and testing. As done in [9,10], we use the H3N2 data for training and H1N1 is withheld for testing. Data was downloaded from the Duke website^a^.

The other three data sets, lung cancer, prostate cancer, and BCell lymphoma, were downloaded from the Kent Ridge Biomedical Data Set Repository^b^. The BCell lymphoma data differs from the others in the following ways. First, it is smaller, both in terms of the number of samples and the number of features. Secondly, there are missing values in the data. We replace missing values with the average expressions for the same gene over all samples. Thirdly, there is no defined distinction between training and testing samples. We randomly withheld 25% of the samples for testing (12 samples of 47 total).

We refer the reader to the original sources, cited in Table 1, for additional details about the data.

### Iterative feature removal

Our Iterative Feature Removal (IFR) process works by repeatedly building a predictive model on training data using a classifier that assigns non-zero weights to only a minimal subset of non-redundant features. In other words, if a gene does not improve the classification accuracy, it is not included in this minimal set. At each subsequent iteration, we remove all features that were selected in previous iterations, limiting the model construction to the remaining features. Thus, we are effectively removing highly discriminatory genes from the data, and forming a new model without these genes. We observe that for a surprisingly large number of iterations, the resulting model is essentially as highly discriminatory. For example, we can remove the best 500 genes whose subsets had accuracies of over 90% on the influenza testing data, and still discover new genes that can classify with essentially *the same* accuracy.

The genes in each set are selected based on their predictive power as a group and we observe that they do not necessarily classify well individually, i.e., they may not provide univariate separation of the data. In contrast, they provide a multivariate model for classifying the data that captures potentially complex biological relationships amongst the variables.

Given the small number of samples in a high-dimensional space, model over-fitting is a concern and must be carefully monitored. At each iteration, we gauge the quality of the model by testing its accuracy on data that was not included in the training. A description of the sparse SVM used for IFR and other methodological details are provided in the Methods section. Results in Additional file 1 illustrate that the IFR procedure can be effective even when using other classification engines.

**Figure 1:**
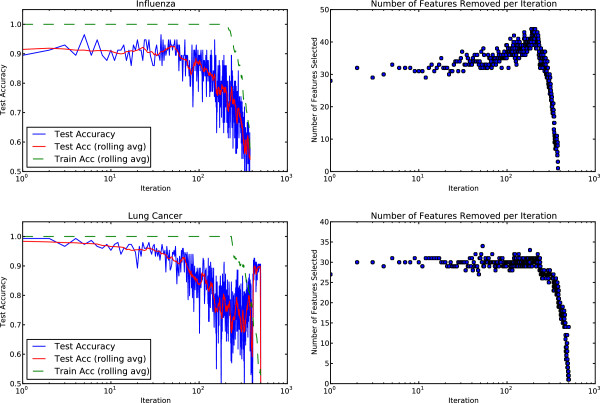
**Iterative feature removal on influenza and lung cancer data.** Iterative Feature Removal is shown using two data sets, influenza (top) and lung cancer (bottom). In each row, the left figure shows the accuracy at each iteration and the right figure shows the number of features selected per iteration. At each iteration, the model is trained without access to any of the genes selected in any of the previous iterations. For the influenza data set there are about 40 sets that are approximately equally predictive identifying approximately 1200 genes. For the lung cancer data there are about 30 sets, or some 900 genes that exhibit predictive properties. The red line represents the rolling average accuracy, illustrating the trend in the data. Figure best viewed in color.

Figure 1 shows the results of applying IFR to the influenza and lung cancer data sets. Additional file 1 contains IFR plots for the prostate cancer and BCell lymphoma data, with the same qualitative characteristics. Additional files 2- 5 provide further details of the results shown in Figure 1. Additional files 6 and 7 provide the genes selected at each iteration when sparse Logistic Regression is used instead of SSVM in the IFR process. The results support our hypothesis that there are many sets of highly predictive features that can be identified in the data beyond the first optimal set identified by a parsimonious model.

Due to the small number of samples, test accuracy from one iteration to the next can vary substantially. This is due, in part, to the fact that the genes are being selected by an optimization criterion on the training data and, as such, this selection is ignorant of the test data. It also suggests that, in general, one should avoid using a hard threshold for stopping the iterative removal process.

The interpretation above is complicated by the fact that when fitting a separating hyperplane to a small number of points in a high dimensional space, there can be many equivalently-optimal partitions. There is thus some element of luck in model selection when validated against withheld data^c^. This effect is less pronounced in the lung cancer data where there are more test samples. When the test accuracy is viewed using a rolling average (red line), the trend becomes clearer.

The training fit (dashed green line) is perfect through the first 200 or so iterations, even as test accuracy varies from trial-to-trial. When the training accuracy is 100% and the test accuracy is low, we infer that the model is over-fit, meaning that the model does not generalize beyond the training data. Genes selected by those iterations showing model over-fitting are not considered strongly discriminative in general, and they are considered to be unrelated to the biology that distinguishes, e.g., symptomatic and asymptomatic subjects in the influenza data.

In the lung cancer data, there is a sharp increase in test accuracy near the end of the iterations. This behavior is explained by the uneven distribution of test samples. Approximately 89% of the test data are from adenocarcinoma (ADCA) tissue samples. After all informative genes have been removed using the IFR process, the training samples can not be accurately fit to the data (green dashed line), even as the test accuracy spikes. In this case, a hyperplane has been constructed such that essentially all points are being classified as ADCA, which results in an artificially high test accuracy.

### More iterations uncover more biology

We have demonstrated that a significant number of feature subsets selected via IFR have essentially equivalent predictive power and hence relate to potentially discriminative biological processes. Our hypothesis suggests that, in many microarray data sets, there is substantial additional biological information contained in the features that remain after the first *optimal* set is removed. Given that genes express in pathways, and can be highly correlated, it is perhaps not surprising that even 20-40 sets can yield highly accurate predictions. While these genes are redundant from the perspective of machine learning, they are essential to the process of biological discovery. Since all the retained sets are predictive, we assert that they play a role in characterizing the differences between the classes in the data.

To evaluate how the resulting larger set of informative genes relates to discriminatory biological pathways, we follow a two-pronged approach, one relying on manual analysis, the other automated. The first approach uses manual expert analysis of the features selected using IFR. The disadvantage of this approach is that it is labor-intensive and thus difficult to apply to cross-validate results. The automated approach relies on employing the Gene Ontology (GO), queried via the GATHER system [14], to identify pathways associated with sets of genes. This approach allows us to design a fully-automated system and to perform repeated trials with cross-validated results. The manual approach takes longer and requires domain expertise, but can yield a more in-depth understanding of the biological significance of the gene subsets, and avoids reporting irrelevant or misleading annotations that the automated system sometimes provides.

There is some similarity between this knowledge-driven analysis and feature enrichment strategies, such as Gene Set Enrichment Analysis (GSEA) [15]. GSEA uses pathway memberships of selected genes to expand the feature set to include additional related genes that were not part of the original selection. In contrast, here we use pathway membership knowledge to group only genes that were selected via IFR, and thus are known to have discriminative power. In the following, we show that grouping genes according to pathways yields powerful classifiers, but these classifiers are not the result of *enriching* the feature selection.

### Manual analysis of genes selected via IFR

We used the influenza 14-16 data for our manual analysis because this allows for direct comparison to other published results.

We examined the 40 most predictive iterations from our IFR process for association with known pathways activated by influenza infection. This number of iterations should not be viewed as a hard cutoff and should be driven to some extent at least by the additional biological information being gained. We made our selection by stopping at the iteration before the consistent decline in test accuracy begins, and where the variance in test results remains relatively low.

As described below, we accumulated related genes across these predictive sets and organized them by pathway. As long as the predictive accuracy of a set is high, the actual iteration that the gene was selected should not be viewed as a strong indicator of its biological importance.

Table 2 lists selected biological pathways we found represented by the set of genes found within the first 40 iterations of IFR. Also shown in the table is the classification accuracy of a model built using only the specified set of genes for each pathway. We find that all pathways yield quality classification results, and several are highly accurate, with accuracies on the H1N1 test data over 90%. Other pathways not listed here may also be present, but an exhaustive identification is not necessary to support our hypothesis.

**Table 2:** Selected pathways from the first 40 iterations of IFR on the influenza data

**Pathway**	**Acc**	**Genes**
Interferon Stimulated Genes	87.7	AIM2 (4), DDX60 (3), GBP1 (1), HERC5 (8), HERC6 (17), IFI27 (2), IFI30 (21), IFI35 (18), IFI44 (1), IFI44L (2), IFI6 (5), IFIH1 (16), IFIT1 (2), IFIT2 (25), IFIT3 (4), IFIT5 (10), IFITM1 (2), IFITM2 (30), IFITM3 (6), IL15 (17), IL15RA (27), IRF7 (13), IRF9 (12), ISG15 (3), ISG20 (34), MX1 (12), OAS1 (1), OAS2 (10), OAS3 (8), OASL (9), PSME1 (5), PSME2 (3), RSAD2 (3), STAT1 (7), STAT2 (27), STAT5B (30), TRIM22 (5), XAF1 (9)
Antigen Recognition Genes	93.0	CD1C (17), HLA-B (27), HLA-DOB (6), HLA-DPA1 (32), HLA-DQA1 (3), HLA-DQB1 (6), HLA-E (26), MICA (22), TAP1 (7), TAP2 (30)
TNF Super Family	89.5	TNF (28), TNFAIP1 (11), TNFAIP3 (29), TNFAIP6 (22), TNFRSF10B (5), TNFRSF14 (31), TNFRSF4 (11), TNFSF10 (11)
IL-1 Beta Receptor Family	86.0	IL1B (14), IL1F5 (12), IL1R1 (10), IL1RAP (6), IL1RL2 (15), IL33 (24)
B Cell Maturation and Activation	91.2	CD19 (36), CD200 (4), CD22 (10), CD24 (7), CD38 (31), CD40 (23), CD72 (28), CD79A (12), CD86 (16), CD9 (7), IGHD (12), IGHM (5), IGHV3-23 (15)
Cell Cycle Related	89.5	CDC20 (13), CDC45L (8), CDCA3 (14), CDCA8 (7), CDK5 (1), CDK5R2 (6), CDKAL1 (5), CDKL5 (27), CDKN1C (13)
Programmed Cell Death	84.2	CASP10 (33), CASP4 (29), CASP5 (24), CASP7 (6), PCDHA3 (14), PCDHGA11 (5), PDCD1LG2 (2), PDCD4 (14)
Chemokines	87.7	CCL11 (19), CCL5 (36), CCR1 (10), CCR10 (4), CCR3 (29), CCR6 (27), CCRL2 (6), CX3CR1 (35), CXCL10 (30), CXCL11 (29), CXCL6 (15), CXCR5 (37), DARC (39)
Cell Adhesion Molecules	87.7	ICAM3 (3), ICAM4 (15), ICAM5 (29), MADCAM1 (25)
Cytokine-Cytokine Receptor Signaling	82.5	IL16 (10), IL17RC (26), IL18RAP (1), IL22 (11), IL9 (31), SOCS1 (33), SOCS3 (29), SOCS6 (9)
Complement Pathway	91.2	C1QA (2), C1QB (3), C2 (19), C3AR1 (26), CR2 (12)
Other Immune Response	93.0	CD8A (22), FCER1G (7), FCER2 (28), FCRL2 (14), KIR2DL3 (32), LY6E (1), LY9 (8), MARCO (26), TLR5 (8)

Selected pathways relating to genes taken from the first 40 iterations of IFR on the influenza data. The iteration that the genes were discovered is in parentheses. Acc indicates the classification accuracy (percent) using only the genes in each list.

**Figure 2:**
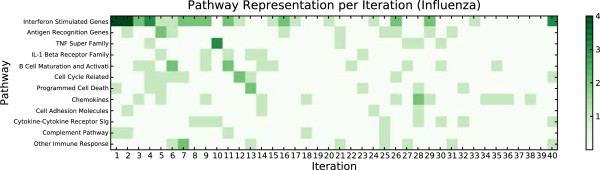
**Distribution of selected pathways over IFR iterations on influenza data.** The Interferon Stimulated Genes comprise the longest list and have representation in half of the iterations. Genes from the other pathways require more iterations to discover. Not all iterations cover the same biological pathways.

Figure 2 shows which pathways were represented by the genes selected at each iteration. No single pathway was represented in all iterations, and no single iteration had representatives from all pathways.

#### Discussion of selected pathways in influenza data

Several markers of the Interferon pathway were found in our analysis (Table 2) and the analysis of Chen et al. [10]. The interferon cytokines (*α*, *β*, *γ*, and *λ*) themselves are not in this subset, nor did we find them to be in our most predictive iterations. That may be in part due to the paracrine, rather than endocrine, nature of these proteins, and that the samples were blood samples, rather than samples directly from the site of infection and viral replication such as the respiratory epithelium. We found many interferon pathway members OAS1-3, OAS-L, PSME1/2, ISG20, IFI35, IFI6, IFI30, IFI16, IFIT2 and IFITM2, for example, all increased in active infection. Interleukin 15 (IL-15) and the IL-15 receptor *α* chain, which are induced by the interferons [16], were also found to increase in symptomatic subjects in our analysis.

Using the 40 most predictive iterations from IFR to look for more biologically relevant pathways activated by infection, we noted two other major cytokine pathways, in particular TNF *α* and IL-1 *β* also played a role. These two proinflammatory cytokines are, like the interferons, well known for their roles in antiviral responses and, in contrast to the interferons, have endocrine effects, notably causing fever [17]. In addition to exploring these pathways, we also noted whether these genes were expressed at higher or lower levels in ill subjects compared to asymptomatic subjects and looked for gene profiles with large differences in expression levels as these would be both useful diagnostically as well as give clear indications of the active pathways. TNF *α* induced proteins TNFAIP6, TNFAIP1, and TNFAIP3 as well as TNF super family 10 (TNFSF10) all increased. Additionally, TNF receptor super family 10B (TNFRSF10B) is the receptor for TRAIL/TNFSF10 but was decreased in symptomatic subjects. Three other TNFRSF members were found to be highly predictive in this analysis, TNFRSF4 (OX40 receptor), TNFRSF9 (CD137/4-1BB), and TNFRSF14 (HVEM), although the expression levels between symptomatic and asymptomatic subjects were only slightly different. All four TNFRSF members are critical for T cell activation and maturation [18].

IL-1 *β*, IL-1 receptor type I, IL-1 receptor accessory protein, and IL-1RL2 were also found to be modulated by infection. IL-1 receptor type I and IL-1 receptor accessory protein decreased in symptomatic subjects, which is somewhat paradoxical as these form the receptor complex for IL-1 *β*. Many B cell genes all decreased upon active infection including IgM heavy chain, IgD heavy chain, CD9, CD19, CD22, CD24, CD72, CD79A, and CD200. This may be due to activation and sequestration of B cells in lymphoid tissues in response to the infection. Similarly, CD8A (primarily expressed on antiviral cytotoxic T lymphocytes) and Ly9 and KIR2DL3 (markers of antiviral natural killer cells) also decreased, suggesting that these cells were also being activated and sequestered in tissues at the site of infection.

Chemokines and their receptors are also important small signaling molecules. CXCL10, which activates chemotaxis in effector T and B increased after infection, but the neutrophil-specific chemokine CXCL6 decreased. CCR1 increased with illness, and is a receptor for many of the CCL chemokines. CCR6, the receptor for CCL20, decreased upon illness. CCRL2 (chemerin receptor) also increased [19]. The fractalkine receptor CX3CR1 increased probably due to trafficking of monocytes [20].

Caspases are involved in apoptotic death of cells. Caspases CASP4, 5, and 7 all increased. Several Programmed Cell Death genes, PDCD1LG2, PCDHGA11, and PCDHA3, also increased in symptomatic subjects. TLR5 increased in symptomatic subjects, unexpected as its most well-known ligand is flagellin. Complement component C2 decreased, but the complement receptors CR2, C3AR1 both increased. Chen et al. [10] had also found C1QA and C1QB as part of their top 50 genes.

Several genes involved in antigen presentation to T lymphocytes were also found to be highly predictive. Chen et al., found TAP1 upregulated in symptomatic subjects, and our analysis also found TAP2. TAP1/2 form a transporter complex to carry peptides into the endoplasmic reticulum for loading into Class I Major Histocompatibility Complex (MHC) proteins. MHC Class I protein/Human Leukocyte Antigen (HLA)-B was also upregulated in symptomatic subjects, along with the related protein HLA-E, which is an activator of NK cells. In contrast, in the Class II chains HLA-DQA1, HLA-DQB1, HLA-DPA1 and HLA-DOB1 were higher in asymptomatic subjects.

#### Combining discriminative pathways

In addition to what has already been described, our hypothesis further suggests that better diagnostic tools can potentially be designed by performing a deeper analysis of the discriminative features selected via IFR. If true, then certain combinations of features from the discriminative pathways should be able to yield classification accuracies that exceed any single iteration because we can improve the signal-to-noise ratio by first selecting discriminative genes and then combining the information from complementary pathways.

As stated in [21], a necessary and sufficient condition for an ensemble to be more accurate than any of its constituent classifiers is when the individual classifiers have predictive power (better than random), and are diverse (they make different errors). Combining the classifiers derived from multiple pathways can, in principle, yield a stronger discriminative model when the pathway-specific classifiers have uncorrelated errors.

To test whether this is true, we combine pairs of pathways and measure the classification accuracy using the influenza data. We generated a Support Vector Machine classifier from each pair of pathways listed in Table 2. For each pair, we combined the features, trained the classifier using the H3N2 data, and measured the classification accuracy using the H1N1 data. With 12 pathways, there are 66 unique pairings. The results of the top 16 are shown in Table 3. The scores for the remaining 50 are available in Additional file 1. By comparison, Table 4 shows previously published classification results on the same data using the same protocol. Several of our single pathway classifiers are competitive with published results, and all of our top-16 pathway pair classifiers are as good or better, providing strong evidence for the benefit of IFR for feature selection.

**Table 3:** Accuracies can improve when combining pathways

**Acc**	**Pathway pair**
100.0	B Cell Maturation and Activation + Cell Adhesion Molecules
98.2	Antigen Recognition Genes + Cell Adhesion Molecules
96.5	IL-1 Beta Receptor Family + Cell Adhesion Molecules
96.5	B Cell Maturation and Activation + Complement Pathway
94.7	IL-1 Beta Receptor Family + B Cell Maturation and Activation
94.7	Cell Cycle Related + Complement Pathway
94.7	Cell Cycle Related + Cell Adhesion Molecules
94.7	Antigen Recognition Genes + B Cell Maturation and Activation
93.0	IL-1 Beta Receptor Family + Complement Pathway
93.0	Complement Pathway + Other Immune Response
93.0	Cell Adhesion Molecules + Other Immune Response
93.0	Cell Adhesion Molecules + Complement Pathway
93.0	B Cell Maturation and Activation + Chemokines
93.0	B Cell Maturation and Activation + Cell Cycle Related
93.0	Antigen Recognition Genes + Other Immune Response
93.0	Antigen Recognition Genes + Complement Pathway

Classification accuracies can improve when combining pathways. We computed the classification accuracies when using all pairs of the pathway gene lists presented in Table 2. Top sixteen results are shown.

**Table 4:** Pathway classification accuracy on influenza compared to other published results

**Acc**	**Classifier**
**100.0**	**B cell maturation and activation + Cell adhesion molecules**
**93.0**	**Antigen recognition genes**
93.0	Bayesian Elastic Net (from [10])
91.2	Bayesian Lasso (from [10])
93.0	Elastic Net (from [10])
91.2	Lasso (from [10])
91.2	Relevance Vector Machine (from [10])
93.0	SVM-RFE (from [10])

In this table, the accuracy of our best single-pathway and best pathway-pair classifiers are compared to the best classifiers reported by Chen et al. [10]. The same protocol is followed: classifiers are trained using H3N2 data from time intervals 14-16 and tested on H1N1 from the same time period.

In searching for discriminative pathways using IFR, we found that we could construct a perfect classifier using the genes identified with B Cell Maturation and Activation (BCell) combined with those identified as relating to Cell Adhesion Molecules (CAM). The classifiers trained from these two pathways make different errors. The BCell classifier misses samples: (9,10,11,17,41), while the CAM classifier misses samples: (1,2,3,4,11,22,33). The only overlap between the two sets of errors is sample 11, but when the two pathways are combined to train a single classifier, no errors are made.

We computed the t-test scores for all genes in the influenza data set. Ranked according to t-test, the 17 genes comprising the combination of the BCell and CAM classifiers are shown in Table 5. Only one is within the top 200 t-test scores, and only five are within the top 500. In fact, all but a few of the pathway and pathway pair classifiers with high discrimination consist primarily of genes with relatively low-ranking t-test scores. (See details in Additional file 1). This highlights the disadvantage of feature selection methods that start by throwing away “low ranking” genes. Doing so prevents the discovery of interaction effects among genes which are not, by themselves, discriminative.

**Table 5:** T-test ranking of genes in the BCell+CAM classifier

**Gene**	**IGHM**	**IGHD**	**IGHV3-23**	**CD200**	**CD24**	**CD9**	**CD22**	**CD38**	**CD19**
**Rank**	831	1238	5659	**122**	**348**	4620	**391**	**337**	508
**Gene**	**CD79A**	**CD86**	**CD40**	**CD72**		**ICAM4**	**ICAM3**	**ICAM5**	**MADCAM1**
**Rank**	**402**	2198	2406	1048		2462	564	4250	8481

The BCell+CAM classifier is 100% accurate on the test data, while consisting of genes that are relatively low ranking in terms of univariate separability. The table shows gene names and the t-test rankings within the total of 12,023 genes (bold values for those within the first 500). A blank cell separates the 13 BCell genes from the 4 CAM genes.

**Figure 3:**
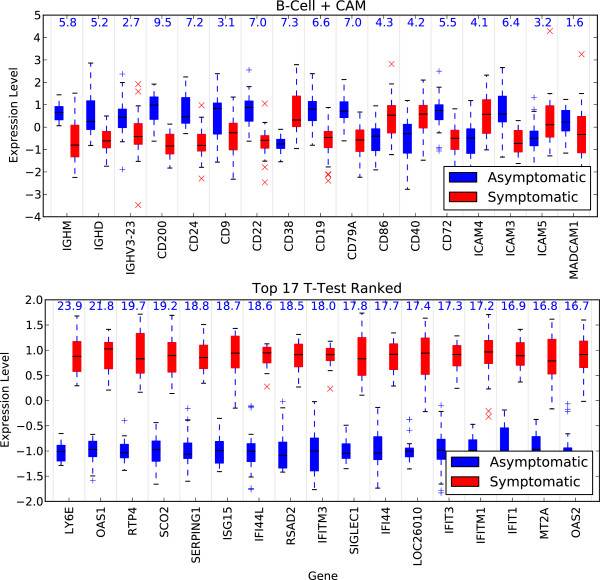
**Univariate separability of discriminative genes.** Boxplots show the gene expression levels (as z-scores) between symptomatic and asymptomatic subjects for selected genes. The first figure shows the expression level for the 17 genes identified by the best pathway pairs classifier (BCell + CAM). The second figure shows the top 17 genes ranked according to univariate t-test scores. The t-test score is provided at the top of the figures for each gene. The genes from the best classifier are far less discriminative in the univariate sense than the top ranked genes, but as a group, they are more discriminative (100% vs. 89.5% classification accuracy). More than two-thirds of the genes from the best classifier fall outside of the top 500 genes ranked according to t-test.

Figure 3 compares the genes from the BCell+CAM classifier to an equal number of the top univariate ranked genes. The accuracy of a classifier constructed from the top 17 genes according to t-test ranking is 89.5%, which is lower than many of the top individual pathway classifiers and lower than all of the top 16 pathway pair classifiers. The t-test statistic score for each gene is shown at the top of the figure in the respective columns. It is interesting to note that the top univariate genes all have higher expression levels for symptomatic patients, while the BCell+CAM genes are mixed.

The weakness in the manual analysis presented herein, including the results of the pathway and pathway pair classifiers, is a lack of cross-validation due to the limits posed by human annotation. We address this limitation by automating our pathway analysis using queries to the well-known Gene Ontology database. These results are presented next.

### Automated analysis of genes selected via IFR

As we did in the previous section, we first present the pathways associated with each iteration of IFR, but this time the pathway annotations are derived from GO using the GATHER web query service. We present the biological significance of features selected beyond the first optimal subset in the lung cancer data. As a reminder, this data consists of two classes of tumors, and so the selected features are those that can be used to differentiate between the two, not to discern whether a tissue sample is cancerous. Following that, we present automated pathway and pathway pair classification using four microarray data sets.

#### Gene ontology annotations

**Figure 4:**
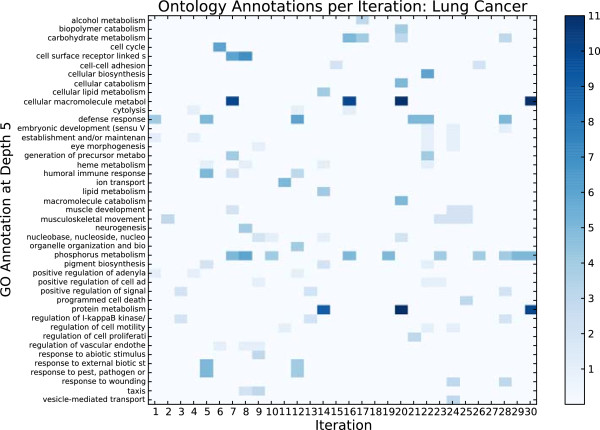
**Gene ontology annotations per iteration on lung cancer data.** The x-axis shows the iteration number, each of which identifies a subset of approximately 30 genes. The y-axis is a set of labels from the GATHER interface to the Gene Ontology, selecting labels at depth 5 in the ontology structure. The intensity of the color at an (x,y) location indicates the number of genes in the subset associated with that label. By comparing one column with another, the results suggest that different iterations contain genes associated with different biological processes. By comparing one row to another, one can see the distribution of genes over iterations relating to a specific process.

The GO annotations associated with the features selected at each of the first thirty iterations are shown in Figure 4. To enhance readability, those labels that are represented by only a few genes across all iterations are not shown. Complete lists of all genes removed at each iteration are available in Additional file 1. While some of the annotations provided via automated queries may be irrelevant, this figure illustrates that different iterations are associated with different subsets of biological factors. There are factors that are common to many of the subsets, yet no single label is present in all iterations.

The annotations in Figure 4 are those from depth 5 in the Gene Ontology structure. The ontology follows a tree-based organization where lower depths are associated with more specific labels. Not all genes are annotated to all depths in the ontology, and thus it is possible to have no annotations at a given depth for a selected set of genes, as observed in the figure for iteration 18. We selected depth 5 for our analysis because it has a balance between label completeness (many genes have depth-5 labels) and specificity (many depth-5 labels are informative). The figure suggests that genes associated with phosphorus metabolism can discriminate between the two types of lung cancer tumors, as this annotation is represented in 10 of the 30 iterations. At the same time, none of the genes known to be associated with phosphorus metabolism were selected within the first six iterations, which covers approximately the first 200 selected genes. Unlike IFR, methods focusing on learning a minimal set of 30-50 discriminative genes (e.g., [4,10]) would be unlikely to designate this pathway as being discriminative.

By considering a larger pool of correlated genes, a biologist might discover important information that otherwise could be masked by feature selection methods optimized for removing predictive redundancy.

#### Discriminative pathways

As we did with the manual analysis, we can use our automated approach to discover discriminative pathways and pathway pairs. Our protocol was to use 50-trial random subset cross-validation on the training partition to identify features associated with the top models. After selecting the top-performing models from the validation stage, we then tested on the withheld data. More details on this procedure can be found in the Methods section.

We also performed the same cross-validation without using multiple IFR iterations, instead using the sparse SVM (SSVM) to select the optimal set of features to classify the data. This is equivalent to using only the first iteration of IFR. The three methods (pathway, pathway pair, SSVM) differ in how genes are selected, but a common classifier engine is used to compare performance. Once the features are identified, validation and test accuracies are computed using a standard (non-sparse) SVM built from the training data using the selected features, and evaluated on the validation or test data. Thus, performance differs due to the features, not the classifier.

Finally, since there can be several models from the validation trials with near equivalent performance, we also report on the average test accuracy of the top five models selected from the validation results.

**Figure 5:**
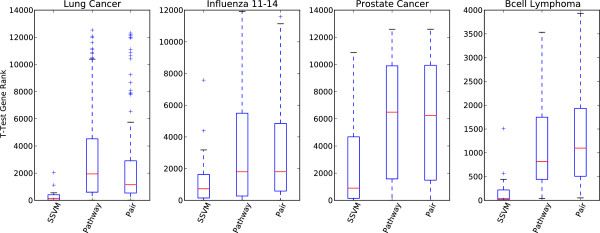
**T-test gene ranking of top classifiers.** The genes from the best model, selected from 50-trial cross-validation, are plotted according to t-test gene ranking using box plots to show the distributions. As with most parsimonious machine learning methods, the sparse SVM (SSVM) classifier tends to select genes which are more univariately discriminative than either the pathway or pathway pair classifiers. Deeper mining of the features using IFR can help identify non-obvious sets of discriminative genes and elucidate discriminative pathways.

Table 6 shows that the pathway and pathway pair classifiers constructed from the genes selected via IFR have discriminative power comparable to the optimal set of genes selected by SSVM, over four data sets. Figure 5 shows that the best pathway and pathway pair classifiers are constructed using genes with generally much lower univariate power (as measured by t-test ranking) than those selected by SSVM.

**Table 6:** Pathway and pathway pair classifiers

		**Single Pathways**			**Pathway Pairs**			**Sparse SVM**		
**Data Set**	**IFR Iters**	**Val.( *μ/σ *)**	**Test**	**Top5**	**Val.( *μ/σ *)**	**Test**	**Top5**	**Val.( *μ/σ *)**	**Test**	**Top5**
Lung	30	95.5/7.0	99.3	96.9	98.8/3.7	98.0	98.8	99.8/1.7	98.0	96.8
Influenza 11-14	30	98.6/2.9	96.0	95.5	99.0/2.3	97.3	97.6	98.9/2.3	92.0	92.8
Prostate	30	78.9/14.2	79.4	77.6	81.8/14.0	79.4	78.2	91.0/5.7	79.4	80.6
BCell Lymph.	20	89.5/12.3	83.3	75.0	91.8/12.4	83.3	78.3	87.8/12.4	83.3	80.0

Cross-validated classification results compared using features selected from discriminative pathways and pathway-pairs, and the “optimal” set of features selected by sparse SVM (SSVM). The latter is equivalent to the first iteration of IFR. The mean and standard deviation of 50 cross-validated trials are shown, followed by the accuracy of the best model from the validation trials applied to withheld test data. Also shown is the average accuracy of the top 5 models on the withheld test data.

In summary, the pathway, pathway-pair, and SSVM feature selections were found to perform comparably on the withheld test data for the lung cancer, prostate cancer, and BCell lymphoma data sets. There was, however, a statistically significant improvement using the pathway-pair features in the influenza data. Significance was measured using Welch’s two-sample t-test between methods, evaluated on the test accuracies returned by the top five models from each. P-values for all comparisons can be found in Additional file 1, with notable results discussed below.

The benefit of using IFR is that several predictive pathways can be identified with performance similar to that of the optimal model, but with the advantage of providing more insight into the biology of the discriminating factors.

In the lung cancer results, there is some evidence that pathway-pair feature selection performs better than SSVM (p-value=0.085), but the p-value is too high to be conclusive. Since all methods perform well, ceiling effects may inhibit the ability to differentiate amongst them.

In the influenza 11-14 results, there was strong evidence that the pathway-pair features yield better classifiers than those selected via SSVM (p-value=0.0002). Additionally, there was some evidence that even individual pathway features perform better than SSVM (p-value=0.056), and that genes selected via pathway-pair analysis outperform those of individual pathways (p-value=0.099). The results of the automated analysis and the manual analysis when applied to the influenza data appear to be consistent. One aspect of the influenza data that is different from the other three is that the training and validation data is of a slightly different pathology (H3N2) than the test data (H1N1).

The results from the prostate data show no statistically-significant differences among the three methods, even though the validation distribution for SSVM has a higher mean accuracy and narrower variance. The features selected by the top performing pathway, pathway-pair, and SSVM model yielded classifiers exhibiting the same accuracy when tested against the withheld test data, correctly classifying 27 of 34 test samples (79.4%). The average test accuracies from the top five models of each method show no statistically-significant difference. With SSVM, the accuracy of the cross-validation result appears to overestimate the performance of the classifiers when applied to withheld data. This phenomenon often indicates that there is a substantial difference in the test data from the training, and that the model is over-fit to the training data. From the description of the data as provided by the Kent Ridge Bio-medical Data Set repository, we note that the test data was compiled from an independent experiment from a different lab with different collection methods. In this case, the pathway-oriented analysis was found to provide more consistent performance (validation to testing) than the parsimonious features selected via SSVM. A similar trend was noted for the influenza data.

There is no statistically-significant performance differences amongst the methods applied to the BCell lymphoma data. The top model from each method, when applied to the withheld test data, correctly classified 10 of 12 samples (83.3%). The BCell lymphoma data only has 12 testing samples, so we observe much higher variance in classification accuracy, where a single misclassification drops the score by over eight percentage points. We note that for all three methods, the top test performance was within one standard deviation from the cross-validated mean. The BCell lymphoma data differs from the rest in that it is smaller, both in terms of samples and features, and it has missing values, which were imputed using averages.

## Conclusions

### Existing feature selection methods

Saeys et al. provides a review of feature selection techniques common in bioinformatics [22], categorizing methods as being filters, wrappers, or embedded. Filters are feature selection techniques that select features without reference to a classifier by employing various statistical analysis techniques. Wrappers are methods that select potential feature sets and then use a classifier to determine the quality of the selected features. Embedded feature selection techniques are those in which the feature selection is intrinsic to the classifier, such as employing *l*_1_ regularization on a linear classifier, resulting in a model built using only a small number of features (those with non-zero weights).

A Support Vector Machine (SVM) is a popular machine learning method that maximizes the width of the margin surrounding a hyperplane that separates classes of data [23]. SVMs have shown promise in classifying samples based on genomic data [2,24,25]. SVMs have also been employed for feature selection using Wrapper and Embedded methodologies. SVM-RFE is an SVM-based feature selection approach that recursively eliminates genes from the data set that are considered lacking in discriminative power [2]. Similar to our method, SVM-RFE also iteratively removes genes, but it removes the least predictive genes until an optimally parsimonious model is obtained, thereby also eliminating *redundant* genes. In contrast, we iteratively remove the *best* features, i.e., most highly discriminatory, in order to demonstrate that there exist many subsets of highly discriminative genes. We assemble all sets of discriminatory genes produced by the iterative removal to form one large set of biologically important genes. Our perspective is that while genes may be redundant in terms of a model’s optimization criteria, they are not necessarily biologically redundant, and may be key to developing a full understanding of the interacting biological processes.

Redundancy has previously been explored in the context of gene expression data. Yu et al. provide formal definitions of “strongly relevant,” “weakly relevant,” and “irrelevant” features [26]. In the context of their discussion, weakly relevant features are redundant with one or more other features. The approach that we advocate in this paper suggests that even weakly relevant features may have key biological implications. Xing et al. discusses Markov Blanket Filtering to iteratively remove redundant features [1]. Yeung et al. points out that there may be equally discriminative gene subsets, and proposes a method to select the top genes by applying Bayesian Model Averaging [4]. However, rather than exploring the totality of these subsets, they employ Bayesian Model Averaging to further condense the set of features to the smallest possible number. As described, their method is limited to finding the 30 best genes. The authors assert the advantage of their method is having high prediction accuracy while using smaller numbers of genes than competing methods on several microarray data sets.

Selecting genes by ranking according to a univariate measure is a common practice among biologists. Significance Analysis of Microarrays (SAM) [27] is a scoring system based on a set of t-test statistics. RankGene [28] computes gene rankings using any of several possible univariate measures, including t-test, information gain, and sum of variances. However, as demonstrated by our results and Additional file 1, genes with limited univariate class separation can have substantial predictive power in a multivariate setting. It is possible that features relating to complex interaction effects can be missed with univariate analysis.

### Broader impact

What is the goal of feature selection on data sets consisting of a small number of samples of microarray data? One goal might be the development of a diagnostic tool. Another goal may be to use the feature selection as an aid in further discovery and learning about a particular biological process.

Regarding the first goal, developing a classifier using a data set with a small number of samples can lead to overly optimistic results. While the classifier may be able to yield high accuracy on the test data, little can be said of how well it could perform as a diagnostic tool in the general population. Imagine developing a decision tree for classifying a notional influenza data set. With only one question: “does the subject have a fever?”, the influenza data could be perfectly partitioned into symptomatic and asymptomatic patients. Some small set of genes could be identified that essentially answer this question. While the selected set of genes may be a perfect classifier on the data set, it would be useless as a diagnostic tool in the clinic, lacking any specificity as to the cause of the fever (flu or otherwise). To restate: identifying a set of features that perfectly separates test data is not the same as identifying a set of features that will diagnose a disease in general. The former only approximates the latter when enough variety is present in the training data.

Lacking a large sample size, using feature selection methods to reduce the decision to a small set of features exacerbates this issue. Training a classifier on a larger set of genes is more likely to lead to the development of a model with greater specificity than training a classifier using a minimum set of genes found to be discriminative in the training data. Consider again the example above, where the classifier learns to partition the data based on the presence of a fever. If instead the classifier were asked to fit a much larger set of genes, then more factors would be involved in the prediction. Instead of generating a false positive for every patient exhibiting a fever but not having the flu, the more complex model would generate false positives only for patients having a larger number of factors similar to the symptomatic training set.

Regarding the second goal, predictive redundancy is not the same as biological redundancy. For example, when considering the host response to a pathogen, there may be several sets of genes which are equally predictive on the test data. One set might relate to the innate immune response (“Process A”), and another set might relate to an acquired immune response (“Process B”). Either set may work for partitioning the test data into symptomatic/asymptomatic subjects. A feature selection technique that suppresses redundant features might eliminate all genes related to Process B without loss of predictive accuracy. Yet when the goal is to select features to generate insight into the biology, the elimination of genes associated with Process B is a problem, not a benefit.

The broader impact of the work presented in this paper is to challenge the paradigm that seeks to identify a small number of discriminative features for microarray data, or other high-dimensional biological data with relatively few samples. Classifiers built in this way are unlikely to be useful as diagnostic tools unless they are trained using a very large number of samples that better approximates the target population for the tool. Feature selection to aid in understanding a biological process based on gene expression data is most useful when a maximally-sized subset of informative genes can be presented to the researcher.

### Summary of significant findings

Iterative Feature Removal provides a process for the unbiased selection of discriminative sets of features from a high-dimensional data set. The procedure results in a deeper mining of relevant information by iteratively suppressing top weighted features in order to unmask the less-obvious features that are nearly equally discriminative.

We recognize that others have previously observed the phenomenon where top genes can be removed from a microarray data set and a subsequent classifier built to similar accuracy using the remaining set (e.g., Gruvberger et al. [29]). However we are the first to present a method for feature selection that leverages this phenomenon as a core mechanism.

When applied to microarray data, IFR identified sets of genes that were highly predictive even when the sets were composed of genes that, taken individually, appear non-discriminatory. Through automated and manual expert analysis of the selected genes, we demonstrated that the selected features exposed biologically-relevant pathways that other approaches would have missed.

The pathway analysis provides an alternative way of grouping the genes, as opposed to by ranking, and also potentially provides more insight to the biologist. By showing that strong classifiers can be built without consideration to the iteration number or any other ranking (other than the fact that we selected the gene via IFR), we prove that strong discriminative power can reside in lower ranking genes.

## Methods

### Sparse linear support vector machine

The model for the classifier is a separating hyperplane

(1) $$f(x)=w^{T}x-b$$

where *x* is a vector of gene expression levels. The *learning* task is to use labeled observations to determine the parameters *w* and *b* such that unlabeled observations can be characterized, e.g., as associated with symptomatic or asymptomatic subjects.

Extending the work of Cortes and Vapnik [23], Mangasarian proposed [30] an arbitrary norm separating hyperplane as the solution to the optimization problem

(2) $$\begin{array}{l} \text{Minimize}∥w{∥}_{p}+C\underset{i}{\sum }\xi_{i}\\ \text{subject to}\\ \;w\cdot x_{i}+b+\xi_{i}\geq 1,\;x\in S^{+}\\ \;w\cdot x_{i}+b-\xi_{i}\leq -1,\;x\in S^{-}\\ \;\xi \geq 0. \end{array}$$

where *S*^+^ and *S*^-^ denote the sets of positive and negative class samples, respectively. The width of the margin separating the two classes is 2/∥*w*∥, which is maximized when ∥*w*∥ is minimized. Slack variables, *ξ*_*i*_, measure the degree of misclassification of samples *x*_*i*_. The parameter, *C*, determines the penalty assigned to the total error from misclassified samples, resulting in a trade-off between the width of the margin and the error penalty.

The *p*-norm of a vector is defined as ${∥w∥}_{p}={\left(\sum_{i}{\left|w_{i}\right|}^{p}\right)}^{1/p}.$ For *p* = 2, we have the standard SVM. For *p* = 1, sparsity is induced in the weight vector *w*. In other words, a separating hyperplane is found where many components of *w* are essentially zero. We refer to the solution of Equation (2) with *p* = 1 as a *sparse support vector machine* (SSVM). The sparsity inducing properties of the 1-norm are now well-known; see [31] and references therein. Our approach follows where the solution to the optimization problem given by Equation (2) is determined using a primal-dual interior point algorithm.

In our gene selection problem, we may interpret sparsity as the elimination of genes that are not essential for classification. This is modeled mathematically by setting the corresponding weight component to zero. For example, if gene *x*_*i*_ co-expresses with another gene *x*_*j*_ then the model will attempt to set either *w*_*i*_ or *w*_*j*_ to zero. Both genes may have high discriminatory value, but they may not both be needed in a single model since they contain similar information. We view one of these genes as a *proxy* for the other.

At each iteration of the IFR process, the SSVM determines a minimal set of genes capable of optimally discriminating the training data using the remaining features. For reasons described above, in general we do not expect genes in the same pathway to be found in the same IFR iteration. Indeed, we see that genes in the same pathway tend to be spread over many sets as they are iteratively removed.

When we have a given set of predictive genes and seek to build a new SVM model, such as when building the pathway-specific classifiers discussed earlier, we no longer use the SSVM since we are not implementing feature selection. In this case we use the *p* = 2 SVM model which is not parsimonious.

### Data pre-processing

For each data set, the source microarray data is normalized to have zero mean and unit deviation for each feature. For the influenza data, the H3N2 and H1N1 data are normalized independently, following what was done in [9,10]. Independent normalization was also employed for the prostate cancer and BCell lymphoma data sets. For the lung cancer data, the test data is normalized using the means and standard deviations of the training data, which is required because the lung cancer test data has a highly-skewed class distribution, while the training data does not.

### Parameter selection

We employed cross-validation on the H3N2 data to select the parameters used by the SVM classifier. We found that the choice of *C* was not sensitive within an order of magnitude, and selected *C* = 1.0. We employed a tolerance value of 0.001 for the convergence of the optimization function. These choices also performed well for the other data sets, so we employed the same parameters for all.

**Figure 6:**
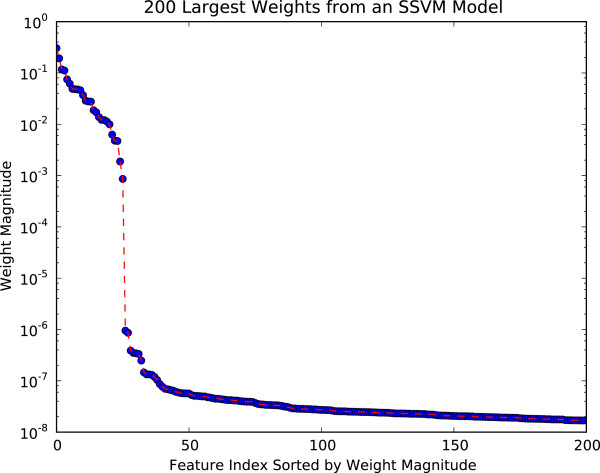
**SSVM weights for first IFR iteration.** Magnitude of top 200 SSVM weights for the first IFR iteration on the influenza data. The cutoff for which weights are set to zero is clearly defined by the steep drop in magnitude.

With SSVM, the weights were determined to be zero by looking for at least a factor of ten drop in the ratio of the weights ordered by magnitude. As shown in Figure 6, the cutoff point where the weights become zero is well-defined. We note that this behavior is not observed in the SVM when a 2-norm is used to minimize the weights in the optimization problem.

### Cross-validation pathway selection

The following provides more detail on how we identified the pathways and pathway pairs in the cross-validated automated analysis, as reported in Table 6.

For each data set, we run 50 trials of IFR on a random 75/25 percent splits of the training/validation data. For each trial, we take the first 30 iterations (20 for BCell lymphoma) to identify the pathways represented by the selected genes using queries to the Gene Ontology. We build a classifier by selecting the genes (without enrichment) representing each pathway and compute the validation accuracy. From one trial to the next, we tend to find the same discriminatory pathways represented, but there is some variation to the representative set of genes. We compute the mean accuracy of a pathway by averaging the pathway classification results over all trials, using zero percent accuracy for any trial where a pathway was not represented. Using these averages over 50 trials, notwithstanding the fact that there is some variation trial-to-trial in which genes represent a pathway, we select the top scoring in terms of mean validation accuracy.

Using the training/validation trials to select a top pathway (or top 5), we then test how well that pathway classifies the withheld test data. We do this by using the union of all the discovered genes relating to that pathway over the 50 trials, creating a new model on the training data, and reporting accuracy on the test.

A similar procedure is followed for pathway pairs. From the same 50 trials we already have the individual pathways. For each trial, we examine the combinations of those pathways that exceed a validation accuracy threshold. The threshold is used to prevent analyzing a combination where one pathway is non-discriminative. This threshold saves computation time, but does not change the results. For each combination, we build a new model on the training data using the union of the genes from the two pathways, and record the validation accuracy of the combination. As we did with the individual pathways, we compute the average validation accuracy of a pathway pair over the 50 trials, using zero percent accuracy for any trial where a pathway pair was not represented. We select the best pathway pairs using the mean validation scores, generate new models on the training data using the union of the genes found by each pair over the trials, and test on the withheld data.

## Endnotes

^a^http://people.ee.duke.edu/~lcarin/reproduce.html

^b^http://levis.tongji.edu.cn/gzli/data/mirror-kentridge.html

^c^ This is not to suggest that random models will classify this data with any accuracy – they don’t.

## Competing interests

The authors declare that they have no competing interests.

## Authors’ contributions

SO and KW provided most of the implementation and analysis presented in this article, with an equivalent technical contribution on IFR. SO developed the pathway classifiers and related analysis, and provided most of the writing for the manuscript as a whole. MK, GH, CO, and MS had the original conceptualization of the iterative removal process, and were involved in subsequent discussions and manuscript editing. MK developed the SSVM implementation used as the sparse classifier for the main IFR results. RS and AS provided the biological expertise for interpreting the nature and relevance of the results. AS performed the manual expert analysis of the selected influenza features and authored the relevant discussion. RS provided editorial support. All authors read and approved the final manuscript.

## Supplementary Material

Additional file 1 **Supplemental Material.** Additional material and analysis is available in the supplemental.pdf file. Click here for file

Additional file 2 **IFR_SSVM_H3N2_genes.** This file is the comprehensive list of genes selected at each iteration when using IFR with SSVM on the influenza data. Click here for file

Additional file 3 **IFR_SSVM_H3N2_acc.** This file provides the accuracy of each iteration of IFR on influenza data using SSVM. Click here for file

Additional file 4 **IFR_SSVM_Lung_genes.** This file is the comprehensive list of genes selected at each iteration when using IFR with the SSVM on the lung cancer data. Click here for file

Additional file 5 **IFR_SSVM_Lung_acc.** This file provides the accuracy of each iteration of IFR on lung cancer data using SSVM. Click here for file

Additional file 6 **IFR_LR_H3N2.** This file is the comprehensive list of genes selected at each iteration when using IFR with sparse Logistic Regression on the influenza data. Click here for file

Additional file 7 **IFR_LR_Lung.** This file is the comprehensive list of genes selected at each iteration when using IFR with sparse Logistic Regression on the lung cancer data. Click here for file
