# Supplementary material for: Iterative feature removal yields highly discriminative pathways
Source: BMC Genomics. 2013 Nov 25;14:832. doi: 10.1186/1471-2164-14-832 (PMC3879090; doi:10.1186/1471-2164-14-832)
Supplement: Additional file 1 — Supplemental Material. Additional material and analysis is available in the supplemental.pdf file. [file 1471-2164-14-832-S1.pdf]

Supplemental

Additional Files

Additional files are provided with this supplemental material. Table 7 lists the supplemental files and provides a description of each.

Table 7: Description of Supplemental Files

File	Description
IFR_SSVM_H3N2_genes.csv	Genes selected at each iteration when using IFR with SSVM on the Influenza data.
IFR_SSVM_H3N2_acc.csv	Accuracy of each iteration of IFR on Influenza data using SSVM.
IFR_SSVM_Lung_genes.csv	Genes selected at each iteration when using IFR with the SSVM on the Lung Cancer data.
IFR_SSVM_Lung_acc.csv	Accuracy of each iteration of IFR on Lung Cancer data using SSVM.
IFR_LR_H3N2.csv	Genes selected at each iteration when using IFR with sparse Logistic Regression on the Influenza data.
IFR_LR_Lung.csv	Genes selected at each iteration when using IFR with sparse Logistic Regression on the Lung Cancer data.

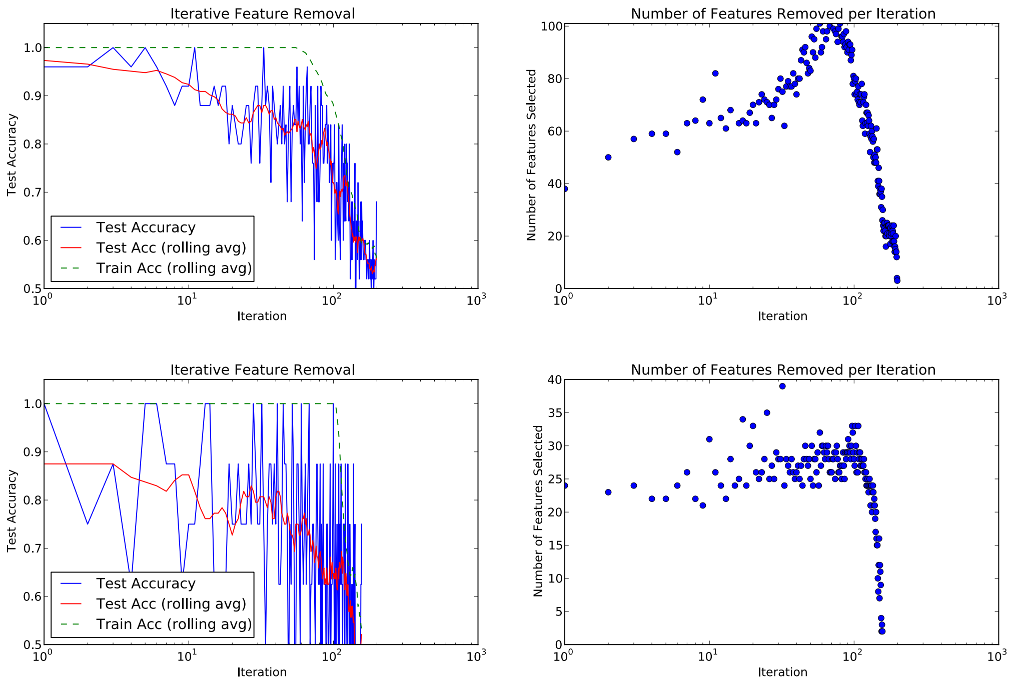

Figure 7: **Iterative Feature Removal on prostate cancer and B-Cell lymphoma data.** The B-Cell data has only 12 withheld test samples, so the test accuracy shows more variation than the other data sets.

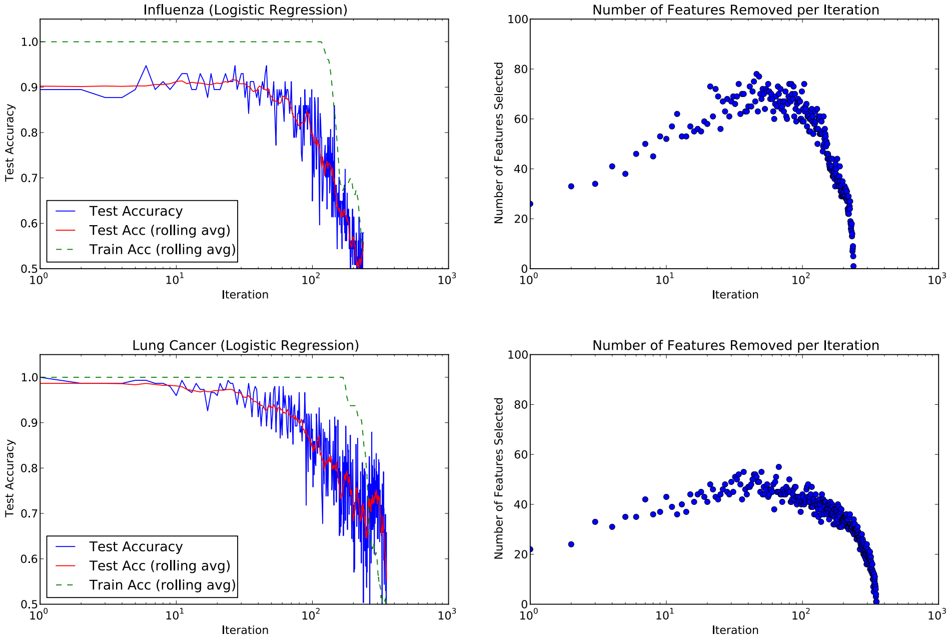

Figure 8: **Iterative Feature Removal using sparse Logistic Regression**, Influenza (top) and Lung Cancer (bottom). This figure illustrates that the choice of sparse classifier used in iterative feature removal does not qualitatively change the results.

Additional IFR Figures

Figure 7 shows IFR applied to the prostate cancer and BCell lymphoma data. The BCell lymphoma data shows more variation in test accuracy in large part because there are only 12 withheld test samples from the complete set of 47.

The choice of sparse classifier does not qualitatively affect IFR results, and thus the conclusions, presented in this article. We show that replacing the SSVM with an $L1$-regularized (sparse) Logistic Regression yields similar iterative removal curves. Figure 8 shows the IFR results using sparse Logistic Regression, which can be compared to the results shown in Figure 1 for SSVM.

KEGG Pathways

We also analyzed the IFR iterations from the lung cancer data using pathways provided by KEGG, the Kyoto Encyclopedia of Genes and Genomes. The KEGG pathway analysis (Figure 9) provides a denser set of labels than GO annotations. Some are metabolic pathways, such as "Calcium signaling pathway," while others are diseases known to be associated with selected genes, such as "Hepatitis C." The KEGG analysis shows that the PI3K-Akt signaling pathway is highly discriminative between the two cancer types, with representation in almost every iteration after the first two. The PI3K-Akt signaling pathway regulates several cellular functions that are critical for tumorigenesis, including cellular proliferation, growth, survival, and mobility [33]. These results suggest that there is a difference in how this pathway is expressed between the two tumor classes.

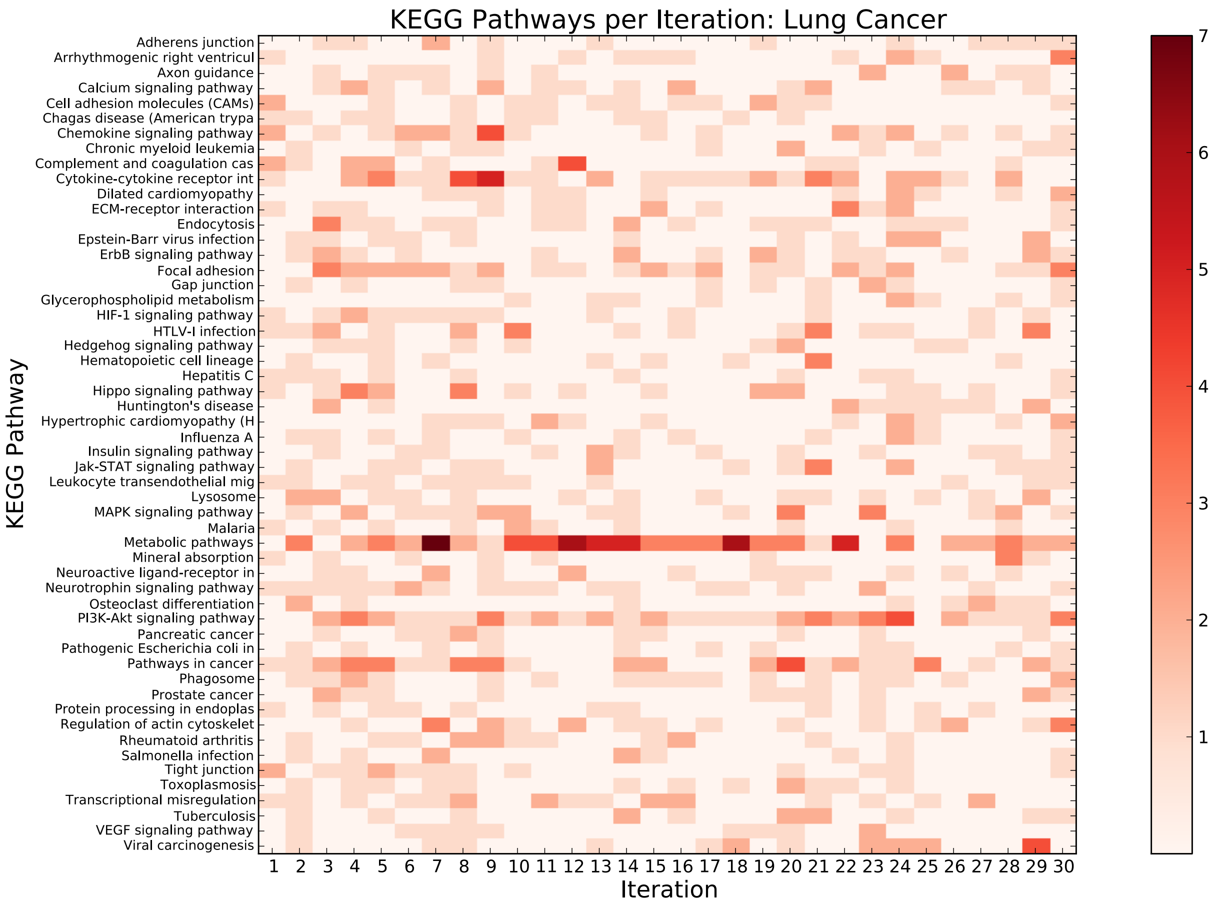

Figure 9: **KEGG pathways per iteration on lung cancer data.**

Limitations to Univariate Analysis

The linear classifiers discussed in this article are multivariate analysis methods. When evaluating the discriminative power of a set of genes, multivariate analysis can capture some of the interaction effects between genes while a univariate analysis can not.

This important point is illustrated in Figure 10. The figure demonstrates the univariate discriminative power (left) and bivariate discriminative power (right) of two genes from the Influenza data. The left sub-figure shows a box plot of the expression levels of the samples from each class for both genes. Red and blue colors indicate class membership, symptomatic and asymptomatic, respectively. The distribution between the two classes for each gene is highly overlapped, and thus neither appears discriminative in the univariate sense. The right sub-figure shows the expression levels in two-dimensional feature space. The green dashed line is the separating line determined by a support vector machine. The circles are the data points, blue and red indicating class membership. The triangles show the same points projected onto each axis, illustrating the significant overlap in the samples when considered in only one dimension. Although the data is not perfectly linearly separable in the bivariate case (5 errors from 51 samples), these genes, when combined, clearly contain significant discriminative power.

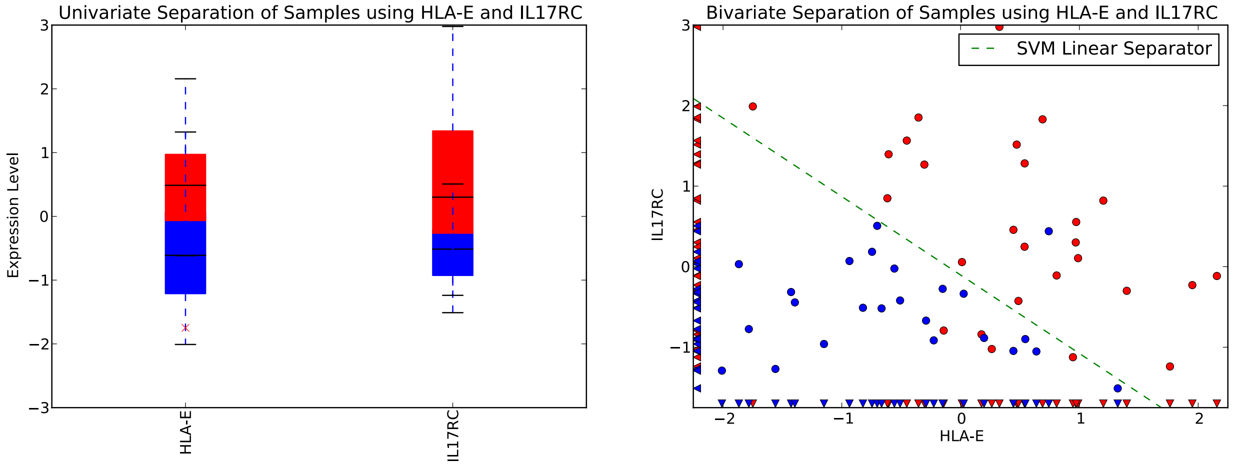

Figure 10: **Discriminatory power of two genes, HLA-E and IL17RC, in the Influenza data.** From univariate analysis (left), neither gene appears discriminative. From bivariate analysis (right), the data can be linearly separated with only a few errors. In the right figure, triangles show the data projected onto each axis.

Univariate Gene Ranking of Discriminative Pathways

Most of the highly discriminative pathways and pathway pairs from Tables 2 and 3, consist primarily of genes that are not within the top 500 genes as ranked by a univariate t-test statistic. Figure 11 shows this trend. For the single pathway classifiers, indexes 2 (Antigen Recognition Genes) and 12 (Other Immune Response), have the highest classification accuracy at 93%. For the pathway pair classifiers, the indexes are sorted in decreasing accuracy. All pathway pairs shown have at least 93% classification accuracy. Top-ranking univariate separability is not required for maximizing discriminative power of a set of genes, and thus is not part of our feature selection strategy.

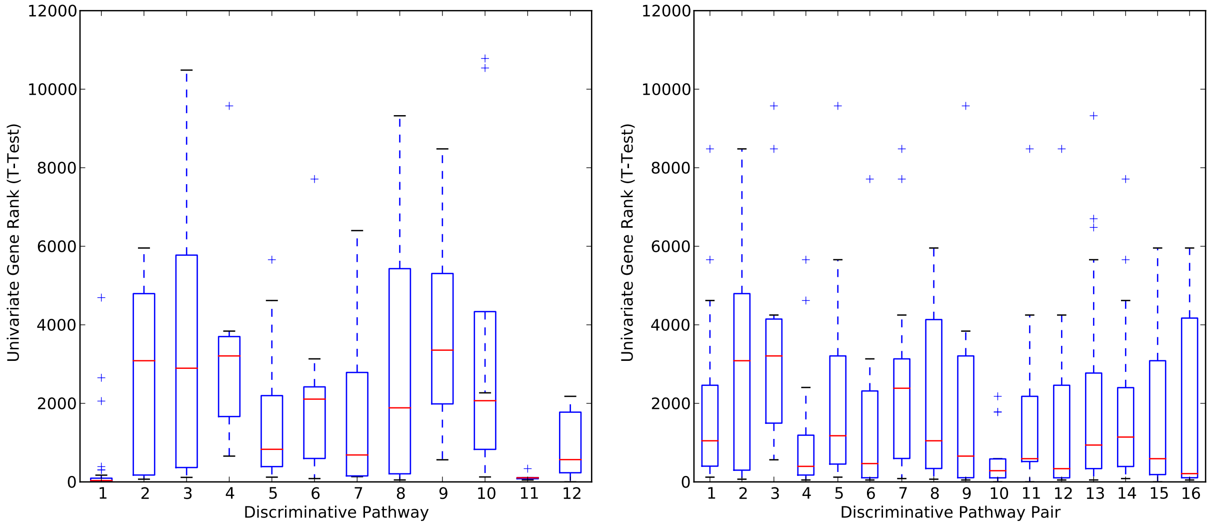

Figure 11: **Gene ranking of genes involved in discriminative pathways.** The figure on the left shows the distribution of t-test based gene ranking for the genes involved in the discriminatory pathway classifiers. The x-axis shows the index of the pathway as listed in Table 2. The figure on the right shows the same information for the top 16 pathway pair classifiers from Table 3.

Additional Discussion: Influenza Pathways

The top 50 genes chosen by Chen et al. [10], are a logical set, primarily composed of interferon induced genes, which have been long known to be primary genes used for antiviral immunity. Their subset also mostly contained genes exhibiting high univariate separation between classes. Indeed, selecting genes that individually exhibit high separability between classes can be useful for identifying diagnostic biomarkers. However, this is not a necessary condition, as interactions between genes can make a set of features highly diagnostic as a group even when, as individuals, they are less so. We found the same genes in our analysis, as well as many other family members and other interferon induced genes, such as IL-15, IL-15RA, ISG20, IFI35, IFI6, IFI30, IFI16, IFIT2 and IFITM2. Our analysis leads to the selection of genes representative of a much wider collection of metabolic processes.

The list of top genes provided by Chen et al. contain only genes that are upregulated in symptomatic subjects, with one exception. While this may only be a coincidental result of their method when applied to the influenza data, there is information that can be learned about the host response to infection from both upregulated and downregulated biomarkers, which our analysis reveals.

Beyond the interferon stimulated genes, several other immunologically-important pathways were found by IFR analysis, and are illustrated by Figure 12. This diagram shows the roles of these gene products in an immune response. As previously described, two other major inflammatory cytokines, IL-1β and TNFα, were found to be highly predictive of symptomatic subjects in the influenza data. These are known regulators of flu symptoms like fever. The TNFα Super Family member, TNFSF10, also known as TRAIL, was also higher in symptomatic subjects.

Not all relevant genes were upregulated in symptomatic patients. This is likely to be the difference in sampling blood populations versus tissues. The receptor complex of IL-1β, IL-1R1 and IL-1Rap, showed reduced expression in symptomatic subjects. CD8A, the alpha chain of the CD8 co-receptor, and the NK cell markers, Ly9/Ly6E, were also reduced in blood leukocytes. This was possibly due to sequestration of these antiviral cells in tissues to fight the infection.

TNFα Receptor Super Family members TNFRSF10B and 9, were also reduced. These were found, along with TNFRSF4 and 14, to be highly predictive between asymptomatic and symptomatic subjects. This

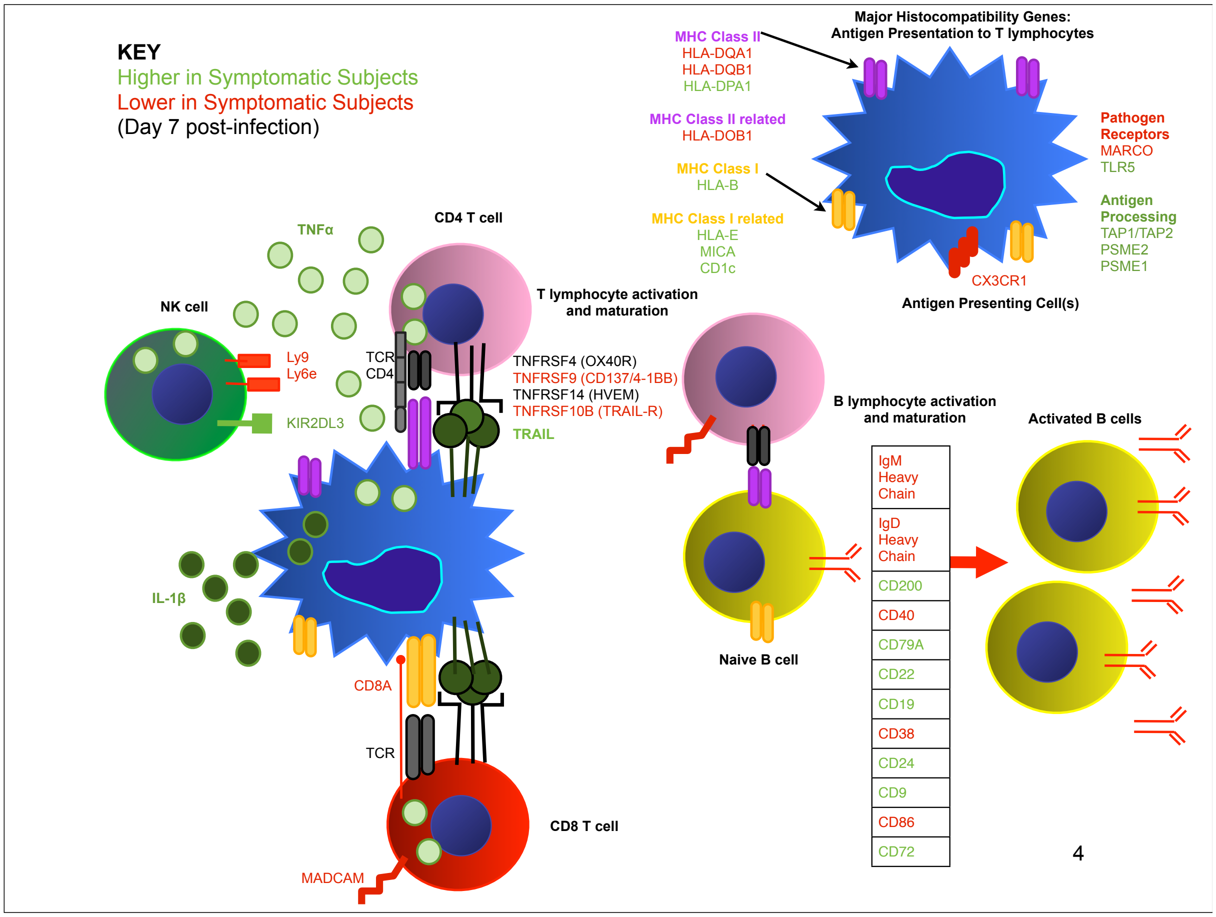

Figure 12: **Discriminative pathways identified by Iterative Feature Removal** on the influenza data. All genes shown in the illustration were discovered within 40 iterations of the IFR process.

family is particularly interesting because several studies have found these genes to be vital for T lymphocyte activation, maturation, and memory in response to influenza infection [34,35].

IGHM and IGHD, the heavy chain genes of IgM and IgD, respectively, are found on naïve B cells, which then class switch to start producing other classes of antibodies. A large number of other B cell markers were also differentially discriminatory between asymptomatic and symptomatic subjects.

Several HLA genes and related members like MICA and CD1c were differentially regulated, most likely on antigen-presenting cells like monocytes, which will differentiate into macrophages and dendritic cells in inflamed tissues. Antigen processing components, like TAP1/TAP2, and PSME1/2 were also upregulated in symptomatic subjects. Pathogen receptors, like MARCO and TLR5 were likewise altered in blood leukocytes of symptomatic subjects. By looking at Figure 12, it becomes more apparent why these would be critical in responding to acute infection by influenza virus.

Complete Pathway Combination Influenza Classification Results

Table 8 provides the classification accuracies when building an SVM classifier from all pairs of pathways identified in Table 2 relating to the Influenza data. Eight pathway combinations (those in bold) yield classifiers that exceed the performance of any single pathway. The highest accuracy attained in any of the iterations of the IFR process on this data was 96.5% (see Figure 1), which was exceeded by two pathway combinations shown in the table.

Table 8: **Classification accuracies of all pathway pairs.** Values in bold face are those combinations that yield classifiers with accuracies superior to the best accuracy of any single pathway. Pathways are: 1: Interferon Stimulated Genes, 2: Antigen Recognition Genes, 3: TNF Super Family, 4: IL-1 Beta Receptor Family, 5: B Cell Maturation and Activation, 6: Cell Cycle Related, 7: Programmed Cell Death, 8: Chemokines, 9: Cell Adhesion Molecules, 10: Cytokine-Cytokine Receptor Signaling, 11: Complement Pathway, 12: Other Immune Response.

Pathway	1	2	3	4	5	6	7	8	9	10	11	12
1	87.7	91.2	87.7	87.7	91.2	86.0	91.2	91.2	89.5	87.7	91.2	89.5
2	–	93.0	87.7	89.5	**94.7**	89.5	84.2	89.5	**98.2**	87.7	93.0	93.0
3	–	–	89.5	89.5	91.2	91.2	84.2	87.7	87.7	86.0	89.5	89.5
4	–	–	–	86.0	**94.7**	91.2	86.0	89.5	**96.5**	84.2	93.0	91.2
5	–	–	–	–	91.2	93.0	87.7	93.0	**100**	89.5	**96.5**	91.2
6	–	–	–	–	–	89.5	84.2	91.2	**94.7**	87.7	**94.7**	87.7
7	–	–	–	–	–	–	84.2	87.7	87.7	84.2	87.7	87.7
8	–	–	–	–	–	–	–	87.7	91.2	89.5	89.5	89.5
9	–	–	–	–	–	–	–	–	87.7	80.7	93.0	93.0
10	–	–	–	–	–	–	–	–	–	82.5	91.2	84.2
11	–	–	–	–	–	–	–	–	–	–	91.2	93.0
12	–	–	–	–	–	–	–	–	–	–	–	93.0

Statistical Significance of Table 6

We employed Welch's two-sample t-test for comparing the performance of the various classifiers on the withheld test data, as reported in Table 6. For each data set, there are three methods, each reporting the accuracy on the withheld data for the top five models selected from the validation results. We computed the t-test between each pair of methods to determine the statistical significance in the performance differences reported by Table 6.

Table 9: Two-sample Welch t-test applied to the top 5 test accuracy scores from the results reported in Table 6. Each entry has the p-value and an indicator (+/-) as to whether the first or second member of the pair had the nominally higher result.

Data	Pathway vs. Pathway Pairs	Pathway vs. SSVM	Pathway Pairs vs SSVM
Lung Cancer	.163 (-)	.926 (+)	.085 (+)
Influenza 11-14	.099 (-)	.056 (+)	.0002 (+)
Prostate Cancer	.855 (-)	.295 (-)	.317 (-)
BCell Lymph.	.667 (-)	.543 (-)	.683 (-)
